# Surgical Treatment of an Aneurysm in the Subacute Stage of Hemorrhagic Moyamoya Disease: Aneurysm Resection Combined With STA-MCA Bypass: A Case Report

**DOI:** 10.31083/RN50491

**Published:** 2026-05-26

**Authors:** Yuezhu Cai, Jiancong Zheng, Jinghui Lin, Jie Wei, Pengyu Lin, Tao Feng, Jiaqi Lin, Haitao Xu

**Affiliations:** ^1^Department of Neurosurgery, Ningbo Key Laboratory of Neurological Diseases and Brain Function, The First Affiliated Hospital of Ningbo University, 315010 Ningbo, Zhejiang, China

**Keywords:** moyamoya disease, cerebral hemorrhage, intracranial aneurysm, aneurysm resection, bypass

## Abstract

**Introduction:**

There is no consensus on the optimal management of intracranial aneurysms in patients with moyamoya disease. Here, we report a case of a patient in the subacute hemorrhagic stage of moyamoya disease with a presumed pseudoaneurysm detected on interval imaging, who was treated with aneurysm resection combined with superficial temporal artery (STA)-middle cerebral artery (MCA) bypass. This report illustrates a feasible individualized surgical approach for similar cases.

**Case Report:**

A 27-year-old male without a prior history of cerebrovascular disease was admitted for a right temporal intracerebral hemorrhage following external ventricular drainage. Initial computed tomography angiography (CTA) at admission showed no aneurysm. One week later, follow-up CTA revealed a newly developed 10 × 9 mm aneurysm in the right MCA region. Subsequent digital subtraction angiography (DSA) demonstrated occlusion of the right MCA M1 segment with the development of characteristic moyamoya collateral networks. The patient underwent microsurgical resection of the aneurysmal lesion, combined with a right STA-MCA bypass. Postoperative imaging demonstrated complete obliteration of the aneurysm, patency of the bypass, and a reduction in moyamoya collateral vessels. Histopathological examination revealed a red blood cell clot surrounded by fibrous connective tissue without definitive evidence of a preserved arterial wall, consistent with a presumed pseudoaneurysm. The patient recovered well, with improved neurological function on follow-up imaging.

**Conclusions:**

In patients with moyamoya disease, a peripheral aneurysmal lesion may become detectable on interval imaging after an initially negative CTA and may represent an unstable hemorrhage-related vascular lesion. In carefully selected cases, lesion resection combined with STA-MCA bypass during the subacute phase may be a feasible individualized treatment strategy, particularly when direct lesion treatment and hemodynamic revascularization are both required. However, longer clinical and imaging follow-up is needed before any firm conclusions can be drawn regarding outcome improvement, or prevention of rebleeding.

## 1. Introduction

Moyamoya disease (MMD) is an uncommon cerebrovascular condition marked by progressive narrowing or occlusion of the terminal portions of the internal carotid arteries, accompanied by the development of an abnormal, fragile collateral vascular network at the base of the brain [1]. The formation and rupture of aneurysms may be additional complications of moyamoya disease [2]. Intracranial aneurysms represent a rare yet high-risk complication of moyamoya disease. The reported incidence of cerebral aneurysms in patients with moyamoya disease ranges from 3% to 15% [3]. Another report indicates that approximately 15% of hemorrhagic events in patients with moyamoya disease are caused by associated aneurysms [4]. Aneurysms arising in moyamoya disease exhibit a far higher hemorrhagic risk than typical unruptured intracranial aneurysms, with reported rupture rates of roughly 70%–87% [2, 5].

Currently, there is no proven pharmacological treatment effective for moyamoya disease. Surgical approaches for moyamoya disease can be classified into three main types: direct bypass, indirect bypass, and combined bypass [6]. However, a unified treatment strategy for intracranial aneurysms in the setting of moyamoya disease has yet to be established [7]. Treatment strategies are usually selected based on the location of the aneurysm, which can be categorized into aneurysms of the circle of Willis and peripheral arterial aneurysms. Aneurysms of the circle of Willis are located on the major arteries of the circle, whereas peripheral aneurysms are found on smaller arteries forming the primitive anastomotic networks of the anterior and posterior circulations [8]. Here, we report a case of hemorrhagic moyamoya disease in which a peripheral aneurysmal lesion became detectable only on interval imaging after an initially negative computed tomography angiography (CTA) and was subsequently managed with lesion resection combined with superficial temporal artery (STA)-middle cerebral artery (MCA) bypass during the subacute stage of intracerebral hemorrhage.

## 2. Case Description

**Fig. 1.**
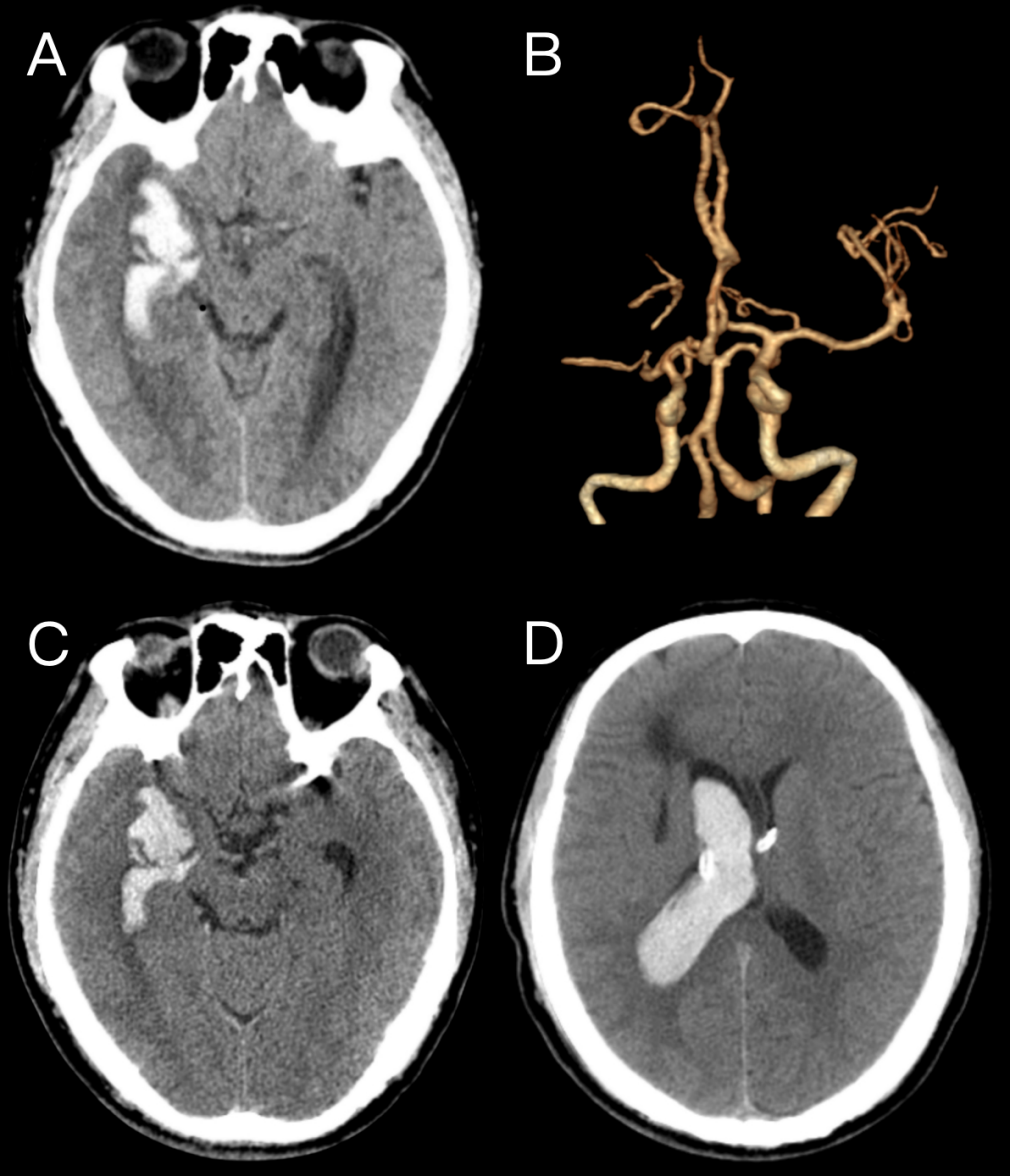
**Imaging manifestations of the local hospital**. (A) Preoperative computed tomography (CT) shows hemorrhage in the right temporal lobe, and the hemorrhage extends to the ventricles. (B) Preoperative computed tomography angiography (CTA) and reconstruction images showed stenosis and occlusion of the M1 segment of the right middle cerebral artery, with no aneurysm observed. (C,D) Postoperative CT scans showed that the hemorrhage focus in the right temporal lobe had recovered and improved.

A 27-year-old male without a known history of cerebrovascular disorders was admitted to a local hospital due to a persistent headache accompanied by a sudden, unexplained loss of consciousness. Emergency cranial computed tomography (CT) showed a right temporal lobe hemorrhage extending into the lateral ventricle (Fig. 1A). CTA demonstrated stenosis and occlusion of the right MCA M1 segment (Fig. 1B). Bilateral external ventricular drainage was performed at the local hospital, and postoperative CT scans confirmed appropriate placement of the drainage catheters (Fig. 1C,D). As the local hospital lacked experience in treating moyamoya disease, the patient was transferred to our institution on postoperative day 6 for further management. The CARE checklist associated with this article can be found in the **Supplementary Material**.

**Fig. 2.**
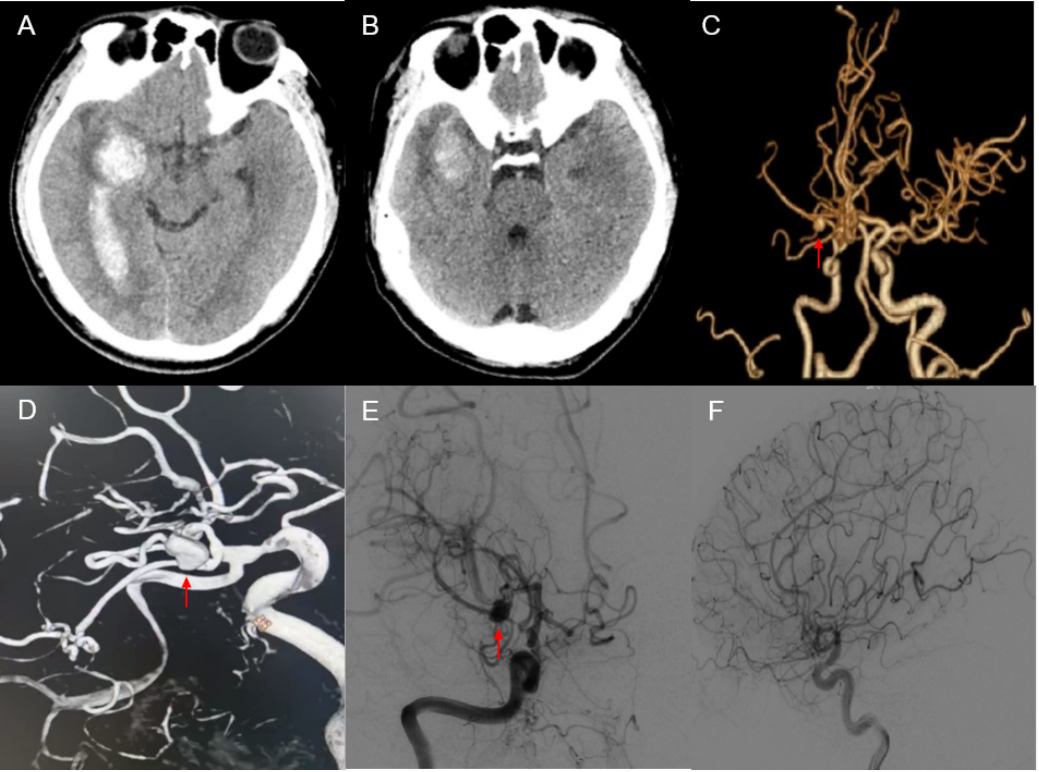
**Preoperative imaging manifestations of our hospital**. (A) One week before the operation, CT showed extensive cerebral hemorrhage foci in the right temporal lobe. (B) CT shows that the hemorrhage focus in the right temporal lobe has shrunk. (C) CTA and reconstructed images show an aneurysm shadow (red arrow) in the right middle cerebral artery area. (D) CTA and reconstructed images show an aneurysm (red arrow) in the right middle cerebral artery area, with a size of 10 × 9 mm. (E) The anteroposterior digital subtraction angiography (DSA) image of the right internal carotid artery showed occlusion (red arrow) of the right middle cerebral artery accompanied by a smoky side branch. (F) The lateral DSA image of the right internal carotid artery shows visible smoke vessels.

Upon admission, the patient was comatose with sluggish pupillary light reflexes and a Glasgow Coma Scale (GCS) score of 1–T–3. CT imaging revealed a right temporal lobe hemorrhage with ventricular dilation (Fig. 2A). After one week of conservative treatment, follow-up CTA revealed a marked reduction in intraventricular hemorrhage, with an aneurysm observed in the right MCA region, measuring approximately 10 × 9 mm (Fig. 2B–D). Follow-up digital subtraction angiography (DSA) confirmed the presence of occluded moyamoya collateral vessels in the right MCA, and the aneurysm was positioned distally relative to these collaterals (Fig. 2E,F).

**Fig. 3.**
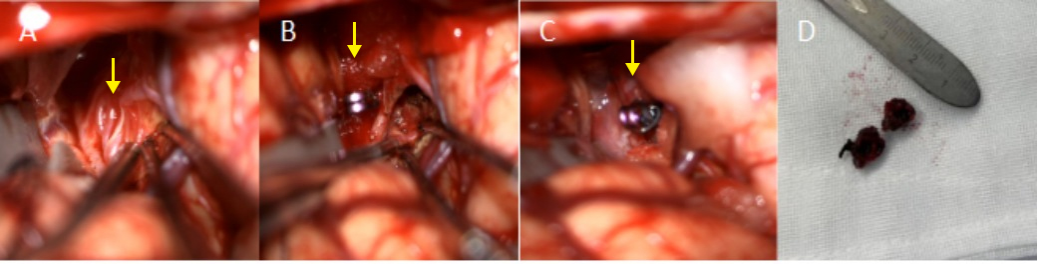
**Intraoperative photo of aneurysm resection**. (A) After the separation of the sylvian fissure, the tortuous and slender moyamoya collateral vessels (yellow arrow) at the origin of the middle cerebral artery can be seen. (B) This aneurysm (yellow arrow) was found in the vascular network of moyamoya disease under the temporal cortex. (C) Use titanium clips to clamp the neck of the aneurysm (yellow arrow) and then remove it. (D) Resected aneurysm.

**Fig. 4.**
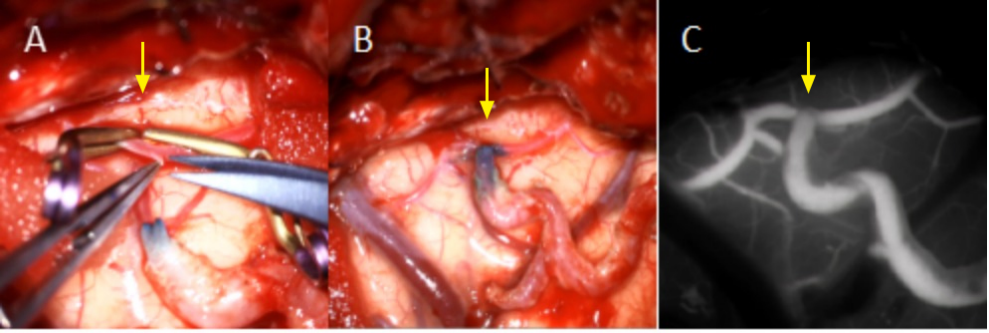
**Intraoperative photo of STA-middle cerebral artery (MCA) bypass**. (A) The middle cerebral artery (yellow arrow) was cut to prepare for anastomosis with the superficial temporal artery. (B) The anastomosis (yellow arrow) between the middle cerebral artery and the superficial temporal artery was completed. (C) Intraoperative infrared thermography was used for observation, and indocyanine green (ICG) angiography confirmed that the bypass graft was patent without obstruction (yellow arrow). STA, superficial temporal artery.

The patient presented with a right temporal lobe hematoma. We opted for aneurysm resection combined with simultaneous STA-MCA bypass. A right frontotemporal craniotomy via a large pterional bone flap was performed. After elevating the bone flap, marked dural tension was observed. To reduce intracranial pressure, cerebrospinal fluid was released by opening the arachnoid membrane of the sylvian fissure. Careful dissection of the sylvian fissure exposed the right MCA M1 segment, where moyamoya vessels were identified (Fig. 3A). An aneurysm was identified in the subcortical region of the temporal lobe (Fig. 3B). The aneurysm neck was clipped, and the aneurysm was subsequently resected (Fig. 3C,D). The superficial temporal artery was then anastomosed to the M4 segment of the MCA, successfully completing the STA-MCA bypass (Fig. 4A–C).

**Fig. 5.**
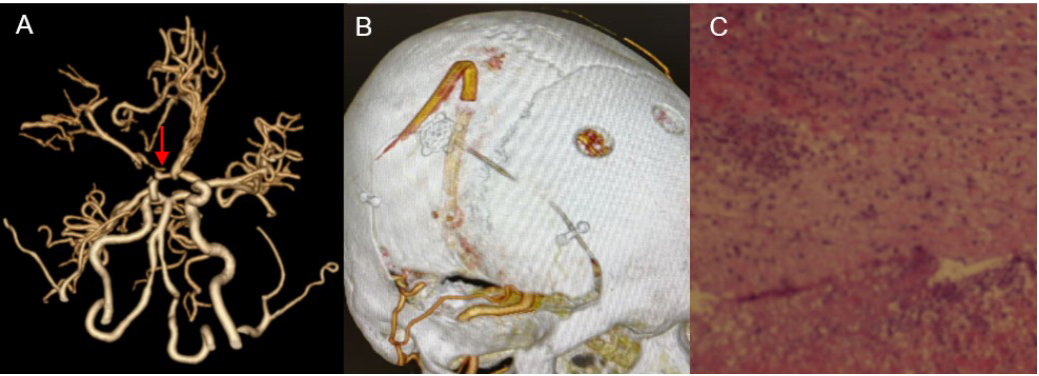
**Postoperative imaging manifestations of our hospital**. (A) CTA and reconstructed images show that the aneurysm has disappeared (red arrow). (B) The 3D reconstructed image shows the bridging position of the STA-MCA. (C) Histopathological examination showed a red blood cell clot surrounded by fibrous connective tissue, without definitive evidence of a preserved normal arterial wall; therefore, the lesion was considered pathologically consistent with a pseudoaneurysm.

Postoperative CTA demonstrated no visible aneurysm in the right MCA region (Fig. 5A). Three-dimensional reconstruction imaging confirmed good patency of the STA-MCA bypass (Fig. 5B). Microscopically, the resected specimen was composed predominantly of an organizing hematoma surrounded by fibrous connective tissue, without a clearly identifiable normal three-layered arterial wall. These findings were considered pathologically consistent with a presumed pseudoaneurysm (Fig. 5C).

**Fig. 6.**
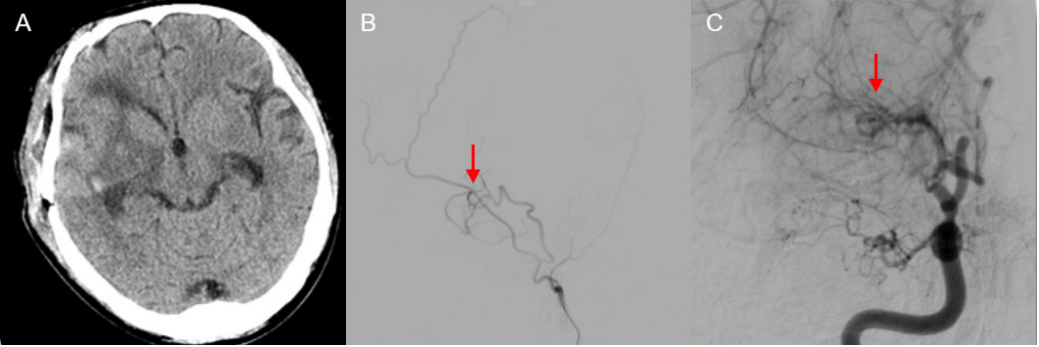
**Imaging manifestations at the follow-up two weeks after the operation**. (A) CT shows that the area of cerebral hemorrhage in the right temporal lobe has recovered and improved. (B) DSA of the right internal carotid artery shows that the collateral vessels on the smoke side have recovered and the bypass vessels have anastomosed smoothly (red arrow). (C) The anterior DSA image of the right internal carotid artery shows that the aneurysm has disappeared (red arrow).

The endotracheal tube was successfully extubated on postoperative day 10. An improvement in the patient’s GCS score to 4-T-6 was noted, and no additional neurological impairments were observed. A follow-up cranial CT obtained two weeks postoperatively demonstrated resolution of the intracerebral hemorrhage (Fig. 6A). Follow-up DSA performed two weeks after surgery demonstrated complete obliteration of the aneurysm, patency of the STA-MCA bypass, and a decrease in the extent of moyamoya collateral networks (Fig. 6B,C).

## 3. Discussion

Intracranial aneurysmal lesions associated with MMD are uncommon but clinically important because of their complex hemodynamic background and hemorrhagic potential. A previous study has shown that approximately 56% of aneurysms are located around the circle of Willis, 18% are found in the basal ganglia region, 22% are distributed along collateral vessels, and a small number are located on other arteries [9]. Among them, aneurysmal lesions involving fragile moyamoya-related collateral vessels are particularly challenging because they are often located distally, may be difficult to access by endovascular techniques, and can arise in the setting of markedly altered cerebral hemodynamics. In addition, lesions related to moyamoya collateral rupture may not always behave like conventional true saccular aneurysms, which makes both diagnosis and treatment selection more complicated. There have been 10 reported cases of peripheral or pseudoaneurysmal lesions associated with moyamoya disease (Table 1, Ref. [5, 10, 11, 12, 13, 14, 15, 16, 17]). The present case is clinically informative because it illustrates the management of a rapidly appearing hemorrhage-related peripheral aneurysmal lesion in a patient with MMD during the subacute stage of intracerebral hemorrhage.

**Table 1.** **Reported cases of pseudoaneurysms and peripheral aneurysms associated with moyamoya disease**.

Authors & Year	Lesion location	Interval imaging	Treatment	Short-term enlargement of the aneurysm	Outcome
Ali *et al*., 2004 [5]	Right LPChA	No	Microsurgical resection	No	Aneurysm disappeared
Ding *et al*., 2023 [10]	Distal AChA	No	Microsurgical resection	No	Aneurysm disappeared
Kanamori *et al*., 2018 [11]	Lateral ventricular wall and left LPChA (2 cases)	No	Combined revascularization surgery	No	The size of the aneurysm has decreased
Lee *et al*., 2023 [12]	Left AChA	No	Indirect bypass surgery by EDAS and observation	No	The size of the aneurysm has decreased
Otawara *et al*., 2007 [13]	Right AChA	No	Bilateral STA-MCA anastomosis and encephaloduroarteriomyosynangiosis	No	The size of the aneurysm remained unchanged
Tsuboki *et al*., 2024 [14]	Distal AChA	No	Super-selective embolization	No	Complete obliteration of aneurysm
Yamada *et al*., 2019 [15]	Right AChA	No	Conservative observation	No	Spontaneous resolution of an aneurysm
Yoon *et al*., 2024 [16]	Left distal LPChA	No	STA-MCA bypass and emergency coil embolization	Yes	Aneurysm disappeared
Yuasa *et al*., 1982 [17]	Left temporal lobe	No	Resection after left frontotemporal craniotomy	No	Aneurysm disappeared
Present case	Right distal MCA collateral vessel	Yes	Microsurgical resection and STA-MCA bypass	Yes	Aneurysm disappeared

AChA, anterior choroidal artery; LPChA, lateral posterior choroidal artery; EDAS, encephalo-duro-arterio-synangiosis; Interval imaging refers to any repeated imaging examination conducted after the initial examination and before the final treatment. In the table, “Yes” indicates that the initial CTA is negative and the lesion is found through interval imaging, while “No” indicates that there is none.

A striking feature of this case was its dynamic imaging evolution. At the local hospital, both preoperative and postoperative CTA failed to identify an aneurysm, whereas follow-up CTA after transfer demonstrated a right MCA-region aneurysmal lesion that was subsequently confirmed by DSA. The interval changes suggested either de novo aneurysm formation after rupture of a fragile collateral vessel or delayed visualization of a previously occult lesion initially masked by the surrounding hematoma. Pseudoaneurysmal lesions differ from true aneurysms in that they are not formed by dilation of an intact trilaminar arterial wall, but rather by focal vessel disruption with subsequent blood collection contained by surrounding fibrous tissue and organized clot [18, 19, 20]. In moyamoya disease, this mechanism is biologically plausible because fragile collateral vessels are exposed to chronic hemodynamic stress and may rupture or undergo wall injury. In the present case, the delayed appearance of the lesion on interval imaging, its location within abnormal collateral circulation, and the pathological finding of a clot with fibrous connective tissue all supported the interpretation of the lesion as a presumed pseudoaneurysm.

Treatment selection in MMD-associated aneurysmal lesions should be individualized according to lesion location, vascular accessibility, hemodynamic status, and hemorrhagic risk. STA-MCA bypass is an important strategy in hemorrhagic MMD because it can improve cerebral perfusion and reduce hemodynamic stress on fragile collateral networks [6, 21, 22, 23, 24]. Although some peripheral aneurysmal lesions have been reported to regress after revascularization alone [23], this process is neither immediate nor reliably predictable. Moreover, pseudoaneurysms may rapidly enlarge and rupture, leading to hemorrhage even after STA–MCA direct bypass [16]. In the present case, the lesion arose within a fragile moyamoya collateral network and became apparent over a short interval in the setting of recent intracerebral hemorrhage, suggesting instability and a potential risk of further bleeding [16, 19]. Therefore, isolated bypass followed by observation was considered less suitable, as waiting for possible spontaneous obliteration might have left the patient exposed to an ongoing short-term risk of lesion enlargement or rebleeding. Endovascular treatment was considered as a potential option [25, 26]; however, DSA demonstrated marked tortuosity and stenosis of the parent vessel proximal to the lesion, making microcatheter and microwire navigation technically difficult. Resection was favored over simple clipping because the lesion was considered a presumed pseudoaneurysmal lesion arising from an abnormal collateral vessel. In addition, microsurgical management permitted direct control of the parent vessel and simultaneous STA-MCA bypass, thereby allowing treatment of the lesion together with correction of the underlying moyamoya-related hemodynamic abnormality in a single-stage procedure. This dual-purpose strategy was an important consideration in the present patient.

The timing of the intervention was another important consideration. Although the optimal timing of revascularization in hemorrhagic moyamoya disease remains controversial [11, 25], the subacute phase may represent a reasonable operative window in selected patients. Compared with the acute phase, cerebral edema and tissue friability may be less pronounced after initial stabilization [27], allowing safer microsurgical manipulation. At the same time, postponing intervention for too long may permit continued exposure of an unstable collateral-vessel lesion to abnormal hemodynamic stress and a potential risk of further hemorrhagic events. In our patient, the surgery was therefore conducted during the subacute phase, after the acute hemorrhagic effects had partially subsided. A staged approach was considered less desirable because it might have increased the overall surgical burden and potentially affected the superficial temporal artery available for bypass. Therefore, combined surgery in the subacute phase was considered a pragmatic and individualized treatment choice.

However, this report has several limitations, including its single-case nature, the lack of pathologically definitive confirmation of pseudoaneurysm, and the limited follow-up period. Therefore, although this case supports the feasibility of combined surgery during the subacute phase in carefully selected patients, firm conclusions regarding long-term durability, outcome improvement, or prevention of rebleeding cannot yet be drawn.

## 4. Conclusions

In patients with moyamoya disease, a peripheral aneurysmal lesion may become detectable on interval imaging after an initially negative CTA and may represent an unstable hemorrhage-related vascular lesion. In carefully selected cases, lesion resection combined with STA-MCA bypass during the subacute phase may be a feasible individualized treatment strategy, particularly when direct lesion treatment and hemodynamic revascularization are both required. However, longer clinical and imaging follow-up is needed before any firm conclusions can be drawn regarding outcome improvement or prevention of rebleeding.

## Availability of Data and Materials

The data that support the findings of this study are available from the corresponding author upon reasonable request.

## Supplementary Material

Supplementary material associated with this article can be found, in the online version, at https://doi.org/10.31083/RN50491.
