# Supplementary material for: Surgical Treatment of an Aneurysm in the Subacute Stage of Hemorrhagic Moyamoya Disease: Aneurysm Resection Combined With STA-MCA Bypass: A Case Report
Source: Rev Neurol. 2026 May 26;81(5):50491. doi: 10.31083/RN50491 (PMC13221674; doi:10.31083/RN50491)
Supplement: Supplementary file 1 [file 1576-6578-81-5-50491-s1.zip › CARE-checklist-English-2013.pdf]

CARE Checklist of information to include when writing a case report

(cc) BY-NC-ND

Topic	Item	Checklist item description	Reported on Line
Title	1	The diagnosis or intervention of primary focus followed by the words "case report"	Page 1
Key Words	2	2 to 5 key words that identify diagnoses or interventions in this case report, including "case report"	Page 1
Abstract (no references)	3a	Introduction: What is unique about this case and what does it add to the scientific literature?	Page 1
	3b	Main symptoms and/or important clinical findings	Page 1
	3c	The main diagnoses, therapeutic interventions, and outcomes	Page 1
	3d	Conclusion—What is the main "take-away" lesson(s) from this case?	Page 1
Introduction	4	One or two paragraphs summarizing why this case is unique (**may include references**)	Page 1-2
Patient Information	5a	De-identified patient specific information.	Page 2
	5b	Primary concerns and symptoms of the patient.	Page 2
	5c	Medical, family, and psycho-social history including relevant genetic information	Not reported
	5d	Relevant past interventions with outcomes	Page 2
Clinical Findings	6	Describe significant physical examination (PE) and important clinical findings.	Page 2
Timeline	7	Historical and current information from this episode of care organized as a timeline	Page 2-4,no timeline
Diagnostic Assessment	8a	Diagnostic testing (such as PE, laboratory testing, imaging, surveys).	Page 2-4
	8b	Diagnostic challenges (such as access to testing, financial, or cultural)	Not reported
	8c	Diagnosis (including other diagnoses considered)	Page 1-2
	8d	Prognosis (such as staging in oncology) where applicable	Page 2
Therapeutic Intervention	9a	Types of therapeutic intervention (such as pharmacologic, surgical, preventive, self-care)	Pages 2
	9b	Administration of therapeutic intervention (such as dosage, strength, duration)	Pages 2
	9c	Changes in therapeutic intervention (with rationale)	Pages 2
Follow-up and Outcomes	10a	Clinician and patient-assessed outcomes (if available)	Pages 2
	10b	Important follow-up diagnostic and other test results	Pages 2–4
	10c	Intervention adherence and tolerability (How was this assessed?)	Not reported
	10d	Adverse and unanticipated events	Not reported
Discussion	11a	A scientific discussion of the strengths AND limitations associated with this case report	Pages 6
	11b	Discussion of the relevant medical literature **with references**.	Pages 3–8
	11c	The scientific rationale for any conclusions (including assessment of possible causes)	Pages 6
	11d	The primary "take-away" lessons of this case report (without references) in a one paragraph conclusion	Pages 6
Patient Perspective	12	The patient should share their perspective in one to two paragraphs on the treatment(s) they received	Not reported
Informed Consent	13	Did the patient give informed consent? Please provide if requested	Yes ☑ No ☐
